# A review on traditional Chinese medicine natural products and acupuncture intervention for Alzheimer’s disease based on the neuroinflammatory

**DOI:** 10.1186/s13020-024-00900-6

**Published:** 2024-02-28

**Authors:** Zhihan Chen, Xinrui Wang, Simin Du, Qi Liu, Zhifang Xu, Yi Guo, Xiaowei Lin

**Affiliations:** 1 School of Acupuncture & Moxibustion and Tuina, Tianjin University of Traditional Chinese Medicine, Tianjin, 301617 People’s Republic of China; 2 School of Traditional Chinese Medicine, Tianjin University of Traditional Chinese Medicine, Tianjin, 301617 People’s Republic of China; 3 Research Center of Experimental Acupuncture Science, Tianjin University of Traditional Chinese Medicine, Tianjin, 301617 People’s Republic of China; 4 Tianjin Key Laboratory of Modern Chinese Medicine Theory of Innovation and Application, Tianjin, 301617 People’s Republic of China; 5 National Clinical Research Center for Chinese Medicine Acupuncture and Moxibustion, Tianjin, 300381 People’s Republic of China; 6 State Key Laboratory of Component-Based Chinese Medicine, Tianjin University of Traditional Chinese Medicine, Tianjin, 301617 China

**Keywords:** Alzheimer’s disease, Traditional Chinese medicine natural products, Acupuncture, Efficacy and mechanism, Neuroinflammation

## Abstract

Alzheimer’s disease (AD) is a neurodegenerative disease with insidious onset and progressive development. It is clinically characterized by cognitive impairment, memory impairment and behavioral change. Chinese herbal medicine and acupuncture are important components of traditional Chinese medicine (TCM), and are commonly used in clinical treatment of AD. This paper systematically summarizes the research progress of traditional Chinese medicine natural products and acupuncture treatment of AD, which combined with existing clinical and preclinical evidence, based on a comprehensive review of neuroinflammation, and discusses the efficacy and potential mechanisms of traditional Chinese medicine natural products and acupuncture treatment of AD. Resveratrol, curcumin, kaempferol and other Chinese herbal medicine components can significantly inhibit the neuroinflammation of AD in vivo and in vitro, and are candidates for the treatment of AD. Acupuncture can alleviate the memory and cognitive impairment of AD by improving neuroinflammation, synaptic plasticity, nerve cell apoptosis and reducing the production and aggregation of amyloid β protein (Aβ) in the brain. It has the characteristics of early, safe, effective and benign bidirectional adjustment. The purpose of this paper is to provide a basis for improving the clinical strategies of TCM for the treatment of AD.

**Graphical Abstract:**

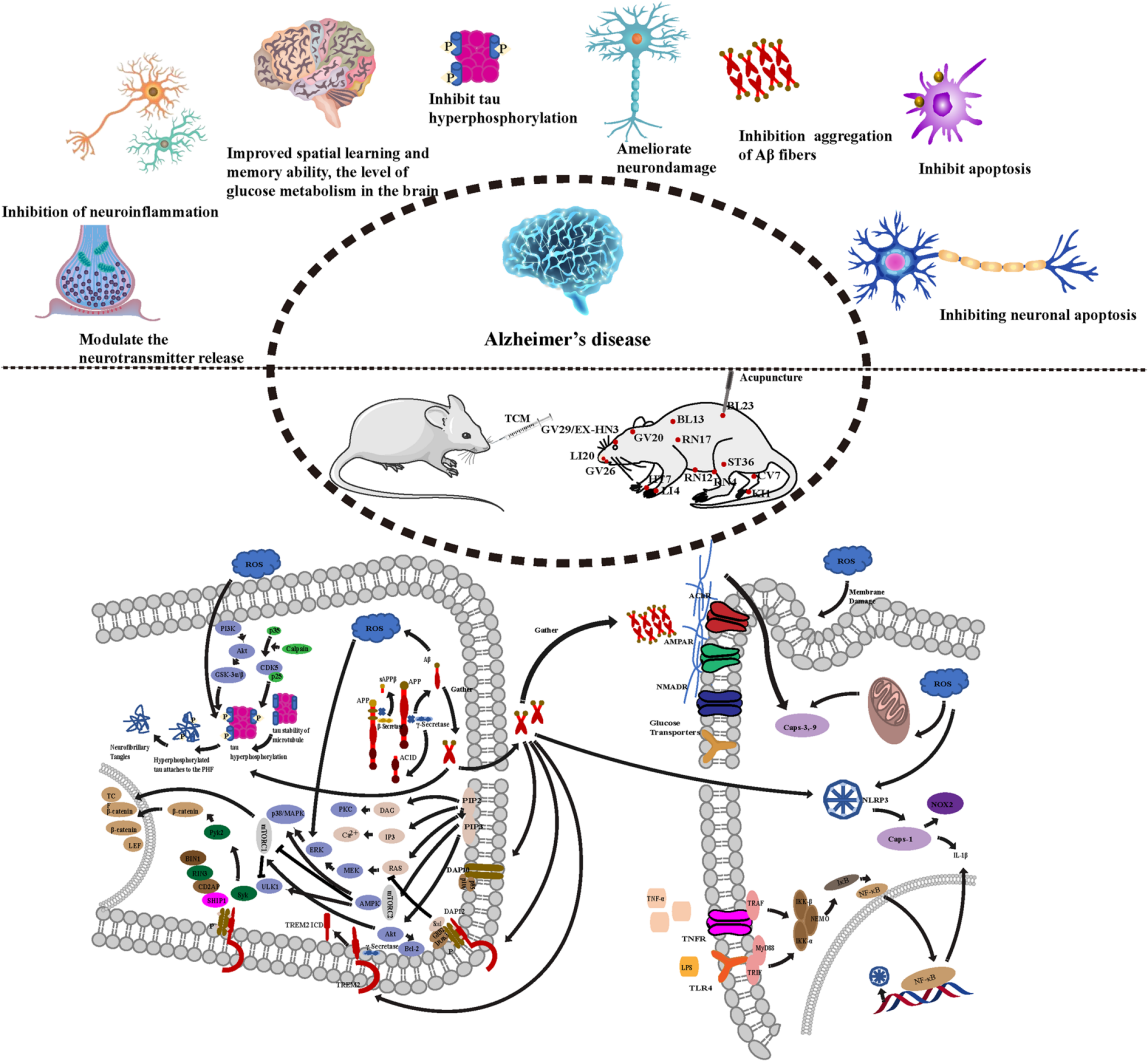

## Introduction

Neurodegenerative disease is a kind of chronic and progressive nervous system disease, which is characterized by the uncontrolled loss of neurons and the accumulation of specific proteins in the brain and spinal cord, accompanied by the decline of brain function, especially cognitive function and motor function [1]. It includes the most common Alzheimer’s disease (AD), Parkinson’s disease (PD) characterized by static tremor, multiple sclerosis (MS) characterized by motor defects, and amyotrophic lateral sclerosis (ALS) characterized by progressive muscle weakness [2]. According to the latest data from the World Health Organization, the number of people living with dementia was around 55 million in 2019 and is expected to rise to 139 million by 2050, according to Word Alzheimer Report 2022. It seriously threatens people's life and health and brings heavy economic burden to society. Different neurodegenerative diseases have similar etiology at the cellular level, such as amyloid aggregation of proteins [3], neuroinflammation [1], oxidative stress [4], mitochondrial dysfunction, neuronal apoptosis [5], etc. However, they have their own unique symptoms. For example, AD is mainly manifested by sensory memory decline and progressive loss of cognitive function [6], while PD is mainly manifested by sensory motor loss [7].

AD is a neurodegenerative disease with occult onset and progressive development [8]. It is clinically characterized by cognitive impairment, memory impairment and behavioral change. Currently, the drugs approved by the U.S. Food and Drug Administration (FDA) for the treatment of AD are symptomatic treatments. These include acetylcholinesterase inhibitors (tacrine, donepezil, galantamine, and carbalatine) [9], the N-methyl-D-aspartate (NMDA) receptor antagonist memantine, and aducanumab, which targets Aβ [10]. Unfortunately, these symptomatic medications are often associated with side effects, including nausea, diarrhea, vomiting, and hypertensive crises. Therefore, developing a safe and effective new drug or therapy for treating AD is very important.

TCM is a traditional medicine originated from China, which has the characteristics of holistic concept and treatment based on syndrome differentiation. It mainly includes internal treatment based on Chinese herbal medicine and external treatment based on acupuncture. Since ancient times, more and more evidence has proved that Chinese herb medicine and acupuncture can effectively improve the clinical symptoms of AD [11–13]. AD belongs to the category of "dementia" in traditional Chinese medicine. The name of dementia first appeared in the Biography of the Divine Doctor Hua Tuo in the Han Dynasty. The key location of the disease lies in the brain and kidney, followed by the spleen, heart and liver. The disease can be divided into two categories: deficiency is caused by Yin deficiency and essence deficiency, brain and pulp insufficiency, and the solid is caused by phlegm turbidness, temperament and blood stasis. Tonifying kidney and essence is the basic principle of TCM treatment of AD, which runs through the whole treatment of AD [14]. The main mechanisms include reducing the production and aggregation of Aβ, inhibiting the phosphorylation of tau protein, inhibiting neuroinflammation, reducing oxidative stress and so on [15]. This paper first summarized the progress of the pathogenesis of AD, reviewed the clinical status of the treatment of AD by TCM, and systematically reviewed and discussed the progress of the therapeutic effect and pharmacological mechanism of effective components of traditional Chinese medicine natural products and acupuncture on AD based on neuroinflammation, in order to provide more reliable evidence for the treatment of AD by TCM.

## Hypothesis of pathogenesis of AD

**Fig. 1:**
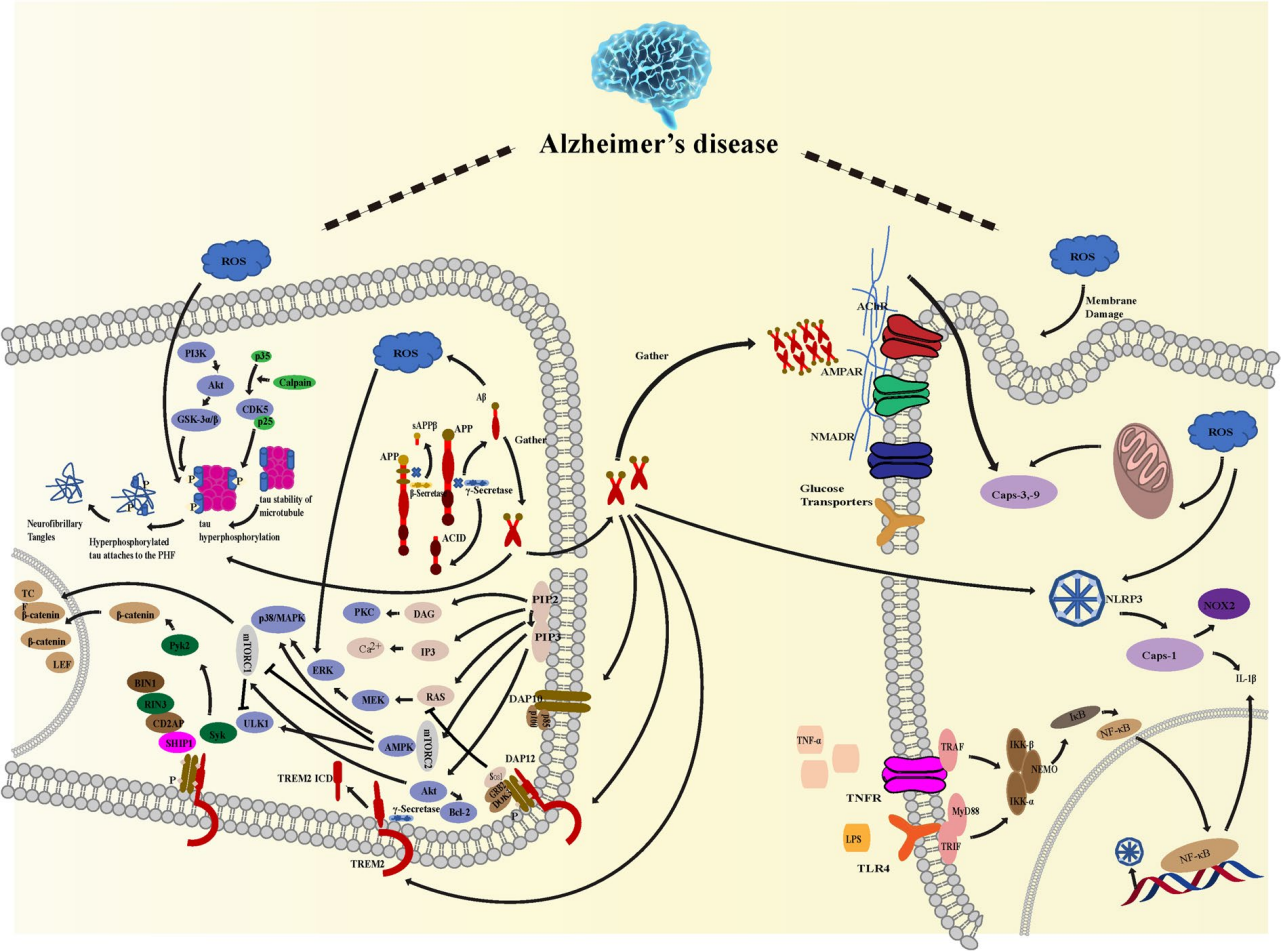
An overview of pathogenesis of AD. (1) In the amyloid-producing pathway, Aβ is produced by the abnormal cleavage of APP by β-secretase 1 (BACE1) and γ-secretase. First, BACE1 cleaved the APP to produce sAPP and C-99 fragments. The C-99 fragment was cleaved by γ-secretase to produce two AICD fragments and an insoluble Aβ fragment. (2) Under normal circumstances, tau can bind to tubulin and stabilize microtubules, playing an important role in maintaining cell polarization, axonal transport and promoting neuronal growth. When tau is over-phosphorylated, tau dissociates from microtubules and aggregates into helical filaments (PHFS), which further aggregates to form NFTs, destabilizing microtubules and ultimately leading to neurofibrillary degeneration. (3) ACh is synthesized by acetyl-CoA and choline and catalyzed by choline acetyltransferase. Acetylcholinesterase in the synaptic cleft terminates signaling by hydrolyzing ACh. (4) Aβ stimulates the activation of microglia and astrocytes and induces reactive gliosis and proinflammatory signaling cascades. Activated microglia and astrocytes reduced Aβ clearance. (5) ROS promote the course of AD mainly through macromolecular peroxidation, Aβ metal ion REDOX potential and mitochondrial dysfunction, and affect cellular homeostasis, free radical production and increase the production of Aβ and p-tau

The main pathologic features of AD are extracellular Aβ plaque deposition and intracellular neurofibrillary tangles (NFTs). The pathogenesis of AD has not been fully elucidated [16]. At present, the pathogenesis of AD mainly consists of hypotheses such as amyloid and tau proteins, neuroinflammation, oxidative stress and cholinergic (Fig. 1).

### β-amyloid and tau hypothesis

The accumulation of Aβ protein and its accumulation and deposition in amyloid plaques are considered as the key pathogenesis of AD [17]. Aβ protein is a transmembrane protein that is produced by hydrolyzing Aβ precursor protein (APP). The first pathological change of AD is mainly the deposition of Aβ protein in the hippocampus [18]. The Aβ protein deposited in the hippocampus and basal segments in the form of amyloid plaques may recruit more Aβ protein to accumulate, thereby forming insoluble aggregates, resulting in mitochondrial damage, disrupting homeostasis, and synaptic dysfunction [19]. Microglia [20] and astrocytes [21, 22] are activated and induce the associated inflammatory response and OS, causing neuronal dysfunction and apoptosis, eventually leading to the development of AD [23, 24]. Tau protein kinase 1 is also activated by Aβ, resulting in abnormal phosphorylation of microtubule-associated proteins and promoting the formation of paired helical filaments (PHFs) and neurofibrillary tangles (NFTs), thereby accelerating the development of AD [25, 26].

Tau protein is a microtubule-associated protein produced by alternative splicing of MAPT gene [27]. The functions of tau protein include maintaining microtubule structure and assisting cytoplasmic transport, maintaining synaptic structure and function, and regulating neuronal signal transduction [28–30]. Tau protein is a phosphoprotein whose phosphorylation and dephosphorylation depend on the balance of protein kinase and protein phosphatase activity [31]. Normally, there are a limited number of tau phosphorylation sites, and they negatively regulate tau binding to microtubules. Under pathological conditions, tau phosphorylation sites may reach saturation, and the highly phosphorylated tau protein in the brain of AD patients may lead to its configuration change and loss of tubulin polymerization ability, resulting in impaired microtubule function [31, 32]. A high level of intracellular tau is involved in tau-tau interactions that may form insoluble PHFs and straight filaments (SFs), resulting in the formation of intracranial fibrous deposits, namely NFTs [33]. NFTs can reduce the number of synapses, produce neurotoxicity, and cause cellular dysfunction [34]. In addition, the acetylation and truncation of tau inhibit its ability to bind to microtubules, promote the occurrence of tau aggregation, and induce the occurrence of mitochondrial dysfunction and synaptic defects [35, 36].

### Neuroinflammation hypothesis

Neuroinflammation is an inflammatory reaction in the CNS characterized by activation of glial cells [37]. Chronic inflammation is a key driver of neurodegenerative diseases, such as AD [38]. Neuroinflammation in AD is mainly driven by microglia (MG) [39, 40], which accounts for 5–10% of all glial cells in the brain, and it belongs to the mononuclear macrophage system. Oligodendrocyte function can maintain and protect the normal function of neurons. When oligodendrocyte is abnormal, it may cause demyelinating disease in the CNS and neuronal damage in severe cases [41]. In this process, astrocytes, oligodendrocytes, neurons, vascular endothelial cells, and peripheral immune cells all participate in the occurrence and progression of neuroinflammation [42, 43]. Neuroinflammation is manifested by the enhanced activation of microglia and astrocytes, increased release of pro-inflammatory cytokines in the brain, increased permeability of blood–brain barrier (BBB), and recruitment of peripheral immune cells into the CNS, eventually leading to neuronal dysfunction. In the case of AD pathology, Aβ stimulates activation of MG and astrocytes and induces reactive glia proliferation and pro-inflammatory signaling cascades. Activated MG and astrocytes reduce Aβ removal. Therefore, inhibiting the occurrence and development of inflammatory reaction is an important strategy for treating neurodegenerative diseases [44].

### Oxidative stress hypothesis

As a key factor of neuronal damage in neurodegenerative diseases, OS is considered as a common contributing factor in a cascade of diseases. OS regulates MS disease within the CNS, and it plays a crucial role in maintaining the development and metabolism of the nervous system [45]. The substances produced by OS are pro-inflammatory substances and they can induce inflammatory responses, which are mainly mediated by OS and ROS. The abnormal aggregation of Aβ protein induces the production of free radicals, and activates ROS to cause oxidative damage and induce OS response. The excessive generation of ROS can result in damage to proteins, lipids, and DNA, leading to irreversible neuronal death [46]. The progression of AD may be mitigated or delayed by inhibiting or reducing OS-induced damage in patients with AD.

### Cholinergic hypothesis

ACh is an important neurotransmitter in the cholinergic system, which is used to maintain the stability of learning and memory processes. AD is caused by the defect of neurotransmitters in the brain of AD patients, which leads to the damage of cholinergic neurons. The decrease of acetylcholinesterase (AChE) and acetylcholintransferase (ChAT) activity is the main reason for the decrease of acetylcholine (Ach) concentration and cholinergic activity, which leads to the decline of cognitive function in AD patients.

In conclusion, the aggregation of Aβ will accelerate the abnormal aggregation of tau protein and the production of reactive oxygen species (ROS). When Aβ accumulates outside the cell to form Aβ plaques, ROS production will be further accelerated, causing OS and resulting in membrane damage. At the same time, the neuroinflammatory response, MG cell activation, cytokine release and astroglial cell proliferation and other complex cascades are induced. In addition, Aβ plaques will destroy synaptic receptors and promote the release of glutamate into the synaptic cleve before synapses. When glutamate is released too much and accumulates in the synaptic cleve or spills over, it will cause the activation of extra-synaptic NMDAR, thus inducing excitatory toxicity.

## Clinical evidence of TCM for AD

By searching Pubmed database, Cochrane, and Clinical trial databases, a total of 17 representative clinical trials related to acupuncture or chinese herbal medicine were screened, including 13 randomized controlled trials (RCTs), 3 before and after controlled trials, and 1 clinical observation. TCM injections or granules were used in 11 cases, acupuncture and moxibustion intervention was used in 3 cases, and acupuncture combined with chinese herbal medicine intervention was used in 3 cases. Based on the existing data, this paper summarized the clinical application status of traditional Chinese medicine and acupuncture in the treatment of AD.

### Clinical status of Chinese medicine in the treatment of AD

At present, neuropsychological scale evaluation is the main method to evaluate the efficacy of dementia drugs, which mainly includes cognitive function, overall function of patients assessed by clinicians, quality of life and mental symptoms [47]. The clinical trial quantified the effect of medication on dementia using neuropsychological assessments at baseline and at the end of treatment [48]. In clinical practice, different neuropsychological measurement scales should be selected according to different evaluation purposes. Now it is widely used in the evaluation scale of cognitive efficacy, including the Mini Mental State Inventory (MMSE), the AD Cognitive Assessment (ADAS cog), and the Montreal cognitive assessment (MoCA) [49]. The concept of activity of daily living (ADL) should be included in the comprehensive functional evaluation, but because of its prominent role in the life of patients [50], the evaluation of ADL has become an essential part of the efficacy evaluation of dementia, which mainly uses the ADL evaluation scale, Very few studies used the caregiver burden questionnaire (CBQ) to evaluate the ability of daily living of patients [51]. A large number of clinical studies have shown that traditional Chinese medicine or integrated traditional Chinese and Western medicine treatment can significantly improve the clinical symptoms and laboratory indicators of Alzheimer's disease in different stages of onset. The chinese medicine used in the treatment of AD mainly has tonifying class, activating blood circulation and removing blood stasis class, phlegm class, mind-awakening class. Di-tan decoction (DTD) [52] is composed of Arisaema Cum Bile, Pinelliae Rhizoma, Aurantii Immaturus Fructus, Poria, Citri Reticulatae Pericarpium, Acori Tatarinowii Rhizoma, Ginseng Radix, Bambusae in Taeniam Caulis, Glycyrrhizae Radix, Zingiberis Recens Rhizoma and dextin. Clinical studies have shown that DTD treatment can increase the ADAS-Cog and C-MMSE scores of AD patients, indicating that it can improve the emotional or cognitive symptoms of AD patients and has a certain safety [52]. Laboratory studies have shown that the memory impairment of AD model mice was significantly reduced by DTD. In the brain tissue in mice treated with DTD, acetylcholine (Ach) and acetylcholine transferase (ChAT) were significantly increased, while acetylcholine esterase (AchE) was decreased. Likewise, Tiaoxin Recipe (TXR), Bushen Recipe (BSR) [53, 54] Jiannao Yizhi Formula [55], Huannao Yicong Formula [56], Fuzhisan [57], Shenfu injection, Shenmai Injectio [58] and other different TCM compounds can improve the scores of ADAS-Cog, CM-SS, MoCA, MMSE and so on.

### Clinical effects of acupuncture on AD

AD is categorized within the realm of dementia in traditional Chinese medicine. According to the principles of traditional Chinese medicine, the kidneys govern bone health and generate vital essence, and it is the sufficiency of this kidney essence that safeguards memory. A deficiency in kidney essence is believed to be the root cause of memory loss. Consequently, Chinese medicine places great emphasis on the approach of “tonifying the kidney and essence” as a primary method for AD treatment [59, 60]. In the context of acupuncture, acupoint selection and technique play pivotal roles in determining treatment efficacy. Manual acupuncture (MA) and electro-acupuncture (EA) represent the two most commonly employed acupuncture methods in clinical practice. Beyond merely stimulating qi through acupuncture, practitioners employ techniques, such as lifting and twisting to manipulate specific acupoints adequately. This approach aims to tonify deficiencies and eliminate excess conditions. For instance, drawing from traditional Chinese medicine principles and clinical experience, TAN Yan [61] identified that acupoints distributed along the 14 meridians, with a concentration in the head and distal extremities, bear significant relevance to dementia treatment [62]. In the case of AD, 24 acupoints are situated across 11 meridians, encompassing points, such as Dumai (GV), Renmai (CV), Pangguangjing (BL), Ganjing (LR), Danjing (GB), Shenjing (KI), Weijing (ST), Pijing (SP), Xinjing (HT), Xinbaojing (PC), and Sanjiaojing (TE). The selection of acupoints for vascular dementia (VD) treatment follows a similar pattern to AD. For VD, the primary 19 acupoints are located along nine meridians, namely GV, CV, BL, LR, GB, KI, ST, SP, and PC. An essential acupoint in kidney tonification is BL23, as it corresponds to the Backshu acupoint of the kidney and plays a crucial role in replenishing kidney essence. Notably, acupuncture at Baihui (GV20) is known for its ability to invigorate the brain, open orifices, and calm the mind. It is widely employed in both research on brain diseases and clinical treatments using traditional Chinese medicine. The combined use of these two acupoints can effectively tonify the kidney, enhance essence, and improve cognitive functions. Contemporary studies have also corroborated that GV20 and BL23 are the most frequently utilized acupoints in research on AD model mechanisms [62]. In a study involving 20 AD patients, a treatment regimen utilizing kidney reinforcement and blood activation acupuncture methods over 12 weeks was employed. Specific acupoints, including Baihui (GV 20), Shenshu (BL 23), Xuehai (SP 10), and Geshu (BL 17), were selected. This treatment was found to enhance the cognitive abilities of AD patients, and its potential mechanism could be associated with the reduction of lipid peroxidation in the brains of AD patients [63]. A clinical study showed that for mild or moderate AD patients, Danzhong (RN 17), Zhongwan (RN 12), Qihai (RN 6), Zusanli (ST 36), Waiguan (SJ 5), and Xuehai (SP 10) were used as the basic acupuncture points, and other acupuncture points were utilized according to patients’ different conditions [64]. On the basis of the comprehensive assessment performed by ADAS-Cog and other scoring systems, acupuncture can improve cognitive function, while the improvement of daily living activities is limited. A study [65] demonstrated that for individuals experiencing early dementia symptoms, a combination of acupuncture at Baihui (GV 20), Sishencong (EX-HN 1), Dazhui (GV 14), Guanyuan (CV 4) points, and the administration of YizhiJiannao Granules yielded superior results compared to the Western medicine group. This was evidenced by improvement in ADAS-Cog and MMSE scores, as well as enhanced blood flow velocity in the middle cerebral artery (MCA), all achieved with fewer associated side effects. Peng [66] demonstrated that combination of acupuncture and moxibustion GV20, EX-HN1, GV14, CV4, and Yizhijiannao Granule significantly improved the effectiveness of the treatment of AD.

Olfactory three needle is a type of acupuncture method, in which the needle of bilateral Yingxiang (LI20) acupoint penetrates inward and upward to the starting point of nasolabial groove, and the third needle penetrates through the nasal root from the one-inch needle of Yintang (DU29). Neuroanatomical studies have revealed that DU29 is situated within the ophthalmic branch of the trigeminal nerve area, while LI20 resides in the upper branch of the trigeminal nerve’s distribution region. Notably, the ophthalmic branch of the trigeminal nerve, specifically the nasociliary nerve, covers the nasal mucosa, including the olfactory epithelium area. This results in an overlapping distribution pattern of both the olfactory and trigeminal nerves. In both clinical and fundamental research settings, the implementation of the Olfactory Three-Needle technique for the management of AD and mild cognitive impairment (MCI) has yielded promising outcomes [67, 68]. Drawing from years of clinical trial experience, Professor Liu Zhibin [69] has concluded that “ Xiusanzhen” can ameliorate cognitive dysfunction in AD patients by acting through the olfactory pathway. Additionally, it significantly enhances learning and memory functions. This effect is believed to be mediated by the reduction of serum endothelin (ET) level, an increase in calcitonin gene-related peptide (CGRP) content, and an overall enhancement of blood circulation in AD patients, thereby leading to improvement in cognitive function.

In summary, clinical research on acupuncture’s role in treating AD has advanced significantly. Acupuncture therapy adheres to well-defined principles within traditional Chinese medicine, concentrating on reinforcing kidney essence, nourishing blood, regulating qi, and awakening the brain. Both manual acupuncture and EA continue to hold essential roles, delivering definite therapeutic benefits. Acupuncture point selection primarily centers around acupoints along the governor vessel, heart channel, pericardial channel, and kidney channel, with clearly defined reinforcement and purgation methods. The parameters for EA, including intensity, amplitude, and frequency, are also well-established. However, it is imperative to note that while the efficacy of acupuncture in treating AD is well-documented, further in-depth research is required to elucidate the specific mechanisms of action underlying these therapeutic effects. This continued investigation will provide a more comprehensive understanding of the treatment’s potential.

In conclusion, the treatment of AD by traditional Chinese medicine is one of the important means to reduce the adverse reactions of western medicine and increase the curative effect [13]. The combination of modern medicine and traditional medicine in the treatment of AD has clinical advantages of increasing curative effect and reducing the toxic and side effects (Table 1). But more high-quality randomized controlled trials are needed to demonstrate the effectiveness of TCM in treating AD.

**Table 1:** Clinical efficacies of integrated TCM and WM for AD treatment

No.	Intervention	Method	Object (T/C)	Disease	Clinical manifestation	Laboratory finding	Refs
1	Di-tan decoction	Randomized controlled trial (RCT)	20/20	Alzheimer’s disease (AD)	Mini-mental state examination (MMSE), Alzheimer’s disease assessment scale cognitive part (ADAS-Cog)	Liver function and renal function	[52]
2	Acupuncture(Baihui (GV 20), Sishencong (EX-HN 1), Zhongwan (CV 12), Wailing (ST 26), Xiawan (CV 10), Qihai (CV 6), Guanyuan (CV 4)) combined with medication(oxiracetam capsules)	RCT	60/60	Vascular dementia (VD) after cerebral infarction	MMSE,ADAS-Cog, clock drawing test (CDT), Barthel index were observed, blood flow velocity of middle cerebral artery (MCA)	Unreported	[65]
3	Abdominal acupoint thread embedding therapy at Zhongwan (CV 12), Xiawan (CV 10), Huaroumen (ST 24), Wailing (ST 26), Daheng (SP 15)	RCT	30/30	AD	MMSE,ADAS-Cog,activity of daily living scale (ADL), neuropsychiatric inventory questionnaire (NPI)	Serum levels of APP and Aβ1-42	[210]
4	Acupuncture Baihui (GV 20), Shenshu (BL 23), Xuehai (SP 10) and Geshu (BL 17)	Before and after	20/0	AD	ADAS-Cog and 8-IPF2alpha concentration in cerebrospinal fluid	Blood and urine before and after treatment were detected by using enzyme linked immunosorbent assay	[63]
5	CMG VS. WMG	RCT	66/65	AD	MMSE, Fuld Object-Memory Evaluation (FOM), Block Design (BD) and Digit Span (DS)	Unreported	[211]
6	Acupuncture at Baihui (GV 20), Sishencong (EX-HN 1), Dazhui (GV 14), Guanyuan (CV 4) + Yizhi Jiannao Granules	Before and after	84/0	AD	MMSE,ADL	Unreported	[66]
7	Tiaoxin Recipe (TXR),Bushen Recipe (BSR)	Before and after	60/0	AD	MMSE,ADL	Unreported	[54]
8	Tiaoxin Recipe (TXR),Bushen Recipe (BSR)	RCT	60/60	Mild cognitive impairment (MCI)	Neuropsychological and N-back	Functional magnetic resonance imaging (fMRI)	[53]
9	Acupuncture RN17(danzhong), RN12(zhongwan), RN6(qihai), ST36(zusanli), SJ5(waiguan) and SP10(xuehai)	RCT	79/0	AD	ADAS-cog,Clinician's Interview-Based Impression of Change-Plus (CIBIC-Plus),Alzheimer’s disease Cooperative Study Activities of Daily Living Scales (ADAS-ADL23) and Neuropsychiatric Index (NPI)	Unreported	[64]
10	Jiannao Yizhi Formula	Clinical observasion	30/30	AD	ADAS-Cog and Chinese Medicine Symptom Scale (CM-SS), MMSE, Montreal Cognitive Assessment (MoCA), and ADL	Serum levels of acetylcholine (Ach), amyloid-β protein 42 (Aβ42), and the microtubule-associated protein tau (Tau)	[55]
11	Fuzhisan	RCT	22/10	AD	ADAS-Cog, NPI	Regional cerebral metabolic rate of glucose consumption (rCMRglc)	[57]
12	Shenfu injection,shenmai injection	RCT	33/33	AD	ADAS-Cog, ADL	Unreported	[58]
13	sage, rosemary and melissa (Salvia officinalis L., Rosmarinus officinalis L. and Melissa officinalis L.; SRM)	RCT	23/22	AD	memory in normal healthy subjects	Unreported	[212]
14	Huannao Yicong Formula	RCT	60/0	AD	ADAS-Cog,CM-SS,MoCA,MMSE	Acetylcholinesterase (AchE) and amyloid-β protein 42 (Aβ42)	[56]
15	Bushenhuatanyizhi, BHY instant granules	RCT	60/0	AD	MMSE,ADL	Superoxide dismutase,lipid peroxide and triglyceride levels	[213]
16	Shenfu injection,shenmai injection	RCT	174/174	Cognitive impairment (CI)	MMSE,MoCA, Chinese medicine (CM),ADAS-Cog,Clinical Dementia Rating (CDR) Total Score, ADL,CM-SS	Serum acetylcholine, acetylcholinesterase, bax, bcl-2,full blood count, kidney and liver function tests, routine urine test and routine stool test	[214]
17	Tianzhi granule	RCT	483/60	Vascular dementia (VaD)	CIBIC-plus	Unreported	[215]

## Potential mechanisms of traditional Chinese medicine natural products and acupuncture for AD

### Potential mechanism of traditional Chinese medicine natural products modulating neuroinflammation in the treatment of AD

Neuroinflammation is the main pathological feature of AD patients. A large number of preclinical studies have found that TCM compounds or monomers can improve the pathological process of neurodegenerative diseases by inhibiting neuroinflammation [70]. Phenolic compounds, flavonoids and terpenoids are the main monomers of traditional Chinese medicine for the clinical treatment of AD. They can reduce neuroinflammation in AD model rats or mice by multi-level, multi-pathway and multi-target, and have the effect of delaying aging and preventing central nervous degenerative diseases. (Table 2).

**Table 2:** Potential mechanisms of TCM natural products for AD

No	Type of compounds	Compound	Chemical structure	Host model	Cellular stress response	Effect of compounds Treatment on the Animal Model	Refs.
1	Polyphenol	Resveratrol		3xTg-AD mice; aluminum chloride (AlCl) and d-galactose(d-gal)-induced AD model mice	Sirt1/miRNA-134/GSK3β; TXNIP/TRX/NLRP3	Reduce toxicity of Aβ oligomers; Suppress of neuronal autophagy; Decrease apoptosis; Inhibit inflammation; Prevent phosphorylation of tau	[80, 81, 83, 85, 216]
2	Polyphenol	Curcumin		ICV-STZ induced AD model rat; MPTP induced PD model mice; LPS induced AD model rat	AMPK; NF-κB; Akt/GSK-3β	Reduce Aβ production; Inhibit Aβ aggregation; Inhibit neuroinflammation; Reduce oxidative damage	[89, 90, 92]
3	Polyphenol	Epigallocatechin gallate (EGCG)		Aβ1-42-induced AD rat model; APP/PS1 transgenic mice	IL-1β,IL-10, IL-13, AchE, Ach	Disrupt Aβ fibril; Reduce Aβ cytotoxicity; Exert neuro-protective effects; Reduce amyloid generation; Diminish the hyperphosphorylation of the Tau protein	[217–219]
4	Flavonoid	kaempferol		I/R model rat; LPS-induced neuroinflammation model mice;	NF-κB, PI3K/Akt, HMGB1 / TLR4	Attenuate neuroinflammation and blood brain barrier dysfunction; Inhibit the microglia pyroptosis	[94, 95, 98, 99]
5	Flavonoid	Quercetin		acute seizure models in mice; Olfactory bulbectomy (OB) animal model; 5xFAD amyloid model mice; SCI model rat; LPS induced rats	AMPK; PI3K/Akt; NF-κB; STAT1	Increase apoE levels; Reduce ROS production; Inhibit Aβ aggregation	[101–103, 220]
6	Flavonoid	Dihydroquercetin		LPS-induced neuroinflammation model mice	IL-6;NO	Inhibit neuroinflammation	[221]
7	Flavonoid	Naringenin		APP/PS1 Transgenic Mic; APPswe / PS1dE9 mice	TNFα, IL1β	Decrease Aβ plaques; Inhibit Aβ aggregation	[109, 110]
8	Flavonoid	Baicalein		BV-2 cell; PD model mice;	TLR4/MyD88/NF-κB; NOX2/STAT1/NF-κB; NLRP3/caspase-1/GSDMD;	Inhibit neuroinflammation	[114–116]
9	Flavonoid	Luteolin		3 × Tg-AD mice	TNF-α, IL-1β, IL-6, NO, COX-2, and iNOS protein	Inhibit ER stress in astrocytes and subsequent neuroinflammation	[222]
10	Flavonoid	Dihydromyricetin		APP/PS1 transgenic mice	NLRP3	Inhibit of NLRP3 inflammasome-based microglia-mediated neuroinflammation	[223]
11	Flavonoid	Icariin		APP/PS1 mice; 3xTg-AD mice	Pyruvate dehydrogenase-E1α (PDHE1α), post synaptic density protein 95 (PSD95)	Reduce hippocampus Aβ deposition; Modulate the differentiation of CD4^+^ T cells; Modulate the release of inflammatory cytokines; Preserve the expression of mitochondrial and synaptic functional proteins	[224, 225]
12	Terpenoid	Ginsenoside Rg1		APP / PS1; Aβ1-42-induced AD rat model; HT22 cells; 3xTg-AD mice	NOX2-NLRP1; NF-κB/NLRP3; CPLX2, SYN2, SNP25	Reduce Aβ deposition; Decrease CDK5 expression; Inhibit PPARγ phosphorylation at serine 273; Decrease NOX2-NLRP1 inflammasome activation and ROS production	[119, 226–228]
13	Terpenoid	Oleuropein		5xFAD model mice; SH-SY5Y cells; APPswe / PS1dE9 mice	NF-κB; RAGE / HMGB1	Inhibit neuroinflammation; Inhibit the formation and aggregation of Aβ fibers	[123, 124]
14	Terpenoid	Oleanolic acid		AD rat model	IL-6, TNF-α, IL-1β	Protect neurons from injury caused by neighboring astrocyte activation	[229]
15	Phenylpropanoids	Verbascoside		APP/PS1 mice	NF- κ B-p65	Inhibit neuroinflammation	[230]

#### Potential mechanisms of polyphenol compounds in the prevention and treatment of AD

In recent years, the role of polyphenols on human health has been paid more and more attention. The main source of polyphenols is vegetables and fruits, which have good antioxidant activity and can effectively scavenge free radicals [71]. As a natural polyphenol compound, resveratrol is easily absorbed by oral administration and excreted in urine and feces after metabolism. A large number of studies have shown that resveratrol [72] has neuroprotective properties, which can improve mitochondrial function, increase the clearance of toxic proteins by CNS, and ultimately improve the spatial learning and memory ability of AD model rats by inhibiting neuroinflammation and direct antioxidant stress [73]. MG and their membrane receptors are key players of neuroinflammation in AD [74]. TLR4/NF-κB/NLRP3 is an important signaling pathway regulating neuroinflammation in microglia [75]. Unactivated NF-κB [76] is usually bound to the inhibitory protein IκB in the cytoplasm. Under the stimulation of Aβ and inflammatory cells, proteasome degradation is accelerated and phosphorylation of IκB inhibitory protein is enhanced, leading to the release and nuclear translocation of NF-κB [77]. With the increase of NF-κB transcription, NLRP3 [78] inflammasome is activated as a multiprotein complex in the cytoplasm. After assembly, the proinflammatory caspase-1 precursor protein is activated into aspase-1, and the inflammatory cytokines IL-1β, IL-6, IL-18 [79] are secreted to induce the immune response of the body. The establishment of a chronic inflammatory environment in the brain can lead to neuronal injury and eventually lead to inflammatory cascade, which further aggravates the course of AD. In vitro experiments showed that in Aβ-induced AD cell model, resveratrol can inhibit TLR4/NF-κB [80]、TXNIP / TRX / NLRP3 signaling and transcription activators [81]. 3xTg-AD mice were created using Psen1 _M146V_ mutation combined with APP_Swe_ and tau_P301L_ genes. It is the closest animal model to familial AD [82]. It has the main neuropathological features of SP and NFT, and the brain shows important pathological changes of AD, such as neuronal death and synaptic loss. In the 3xTG-AD animal model, resveratrol decreased the neuroinflammation and accumulation of Aβ oligomers, increased the levels of neurotrophin, synaptic markers, silencing information modulators, and decreased the markers of apoptosis, autophagy, endolysosomal degradation, and ubiquitination in the brain of 3xTG-AD mice [83]. Silent type information regulation 2 homolog1 (Sirt1) plays an important role in regulating cell stress, metabolism, growth, aging and apoptosis [84]. SIRT1 can reduce the content of Aβ by increasing the proportion of α-secretase of APP or decreasing BACE1 activity. In aluminum chloride (AlCl) and d-galactose (d-gal)—induced AD model mice, it was found that resveratrol can also increase the growth of neurites by activating SIRT1 and inhibiting the expression of microRNA-134 [85]. Through the regulation of these pathways, the overactivation of microglia can be inhibited to play a neuroprotective role.

Curcumin is a kind of yellow small molecule plant polyphenol extracted from the rhizome of turmeric, Curcuma, turmeric, etc. Numerous studies have provided experimental evidence for curcumin in the treatment and prevention of neurological diseases [86]. Curcumin can promote a series of neuroprotective responses by regulating various signal cascades in the brain, such as improving neuronal vitality, promoting neuronal differentiation and inhibiting neuronal apoptosis [87]. This involves a variety of mechanisms, including effects on neurotransmitters in the brain, modulation of the hypothalamic-pituitary-adrenocortical axis [88], upregulation of neurotrophic factor levels, or increased nerve regeneration. In ICV-STZ-induced AD rat model, curcumin can improve neuroinflammation and play a positive role in improving recognition memory [89]. In vivo neuroinflammation model, microglia were overactivated by LPS stimulation, curcumin enhanced AMPK activation in brain regions, and glial fibrillary acidic protein (GFAP), an astrocyte marker, was significantly reduced [90]. In the brain of AD patients, metals can induce amyloid aggregation and toxicity. Therefore, metal chelation can reduce the neurotoxicity caused by amyloid aggregation and oxidation. One Cu^2+^ or Fe^2+^ can bind at least two molecules of curcumin, suggesting another mechanism by which curcumin acts on AD [91]. Due to its binding function to REDOX metals, curcumin can also inhibit the inflammatory damage caused by the NF-κB [92] pathway caused by metals.

#### Potential mechanisms of flavonoid compounds in the prevention and treatment of AD

Plant-derived flavonoids have a wide range of anti-inflammatory and antioxidant activities and can effectively block toxic pathways associated with neurodegenerative disease pathology [93], including kaempferol, quercetin, baicalin and so on.

Oxidative stress is also a kind of important pathological mechanism, in the classic of AD induced by LPS model, contains galangal phenol drugs can significantly improve the activity of catalase, thereby inhibiting oxidative damage in cells, at the same time it also helps to improve alpha-synuclein pathogenic protein expression and tyrosine hydroxylase exception [94]. In spinal cord injury (SCI) model, gavage administration of kaempferol can improve the recovery of hindlimb motor function and spinal cord injury after SCI [95]. In addition, kaempferol administration decreased microglial activation and oxidative stress levels in the spinal cord. In vitro studies showed that kaempferol inhibited LPS-induced microglial activation in BV-2 cells [96]. BV2 cells pretreated with kaempferol reduced reactive oxygen species (ROS) production by inhibiting NADPH oxidase [97], followed by inhibiting phosphorylation of p38 MAPK and JNK, and subsequently inhibiting nuclear translocation of NF-κB p65 to inhibit proinflammatory factor expression [95]. Kaempferol can inhibit NF-κB, PI3K/Akt [94, 98], HMGB1/TLR4 [99]and other inflammatory pathways to play a neuroprotective role in LPS-induced neuroinflammation.

Quercetin is widely found in flowers, leaves, buds, seeds and fruits of many plants, mostly in the form of glycosides, such as rutin, quercetin, hyperin and so on. Quercetin can be obtained by acid hydrolysis. Many studies have reported that quercetin has therapeutic potential for brain diseases [100]. Quercetin treatment has been found to have anticonvulsant activity, which is correlated with brain concentration [101]. In addition, quercetin has been reported to promote antidepressant effects by inhibiting antioxidant effects [102]. What is more, quercetin treatment has been reported to attenuate hypothalamic–pituitary–adrenal (HPA) axis dysregulation in a mouse model of mild traumatic brain injury [102]. Importantly, oral quercetin decreased insoluble Aβ levels in the cortex of amyloid transgenic mouse models [103]. Macrophage polarization plays essential and diverse roles in most diseases. Homeostasis dysfunction in M1/M2 [104] macrophage polarization causes pathological conditions and inflammation. Neuroinflammation is characterized by microglial activation and the concomitant production of pro-inflammatory cytokines, leading to numerous neurodegenerative diseases and psychiatric disorders. In the study, we found that quercetin effectively inhibited the expression of lipocalin-2 in both macrophages and microglial cells stimulated by lipopolysaccharides (LPS). The production of nitric oxide (NO) and expression levels of the pro-inflammatory cytokines, inducible nitric oxide synthase (iNOS) and cyclooxygenase (COX)-2, were also attenuated by quercetin treatment [105]. The results also showed that quercetin significantly reduced the expression levels of the M1 markers, such as interleukin (IL)-6, TNF-α, and IL-1β, in the macrophages and microglia [106]. In addition, quercetin markedly reduced the production of various ROS in the microglia [107]. The microglial phagocytic ability induced by the LPS was also effectively reduced by the quercetin treatment. Importantly, the quercetin increased the expression levels of the M2 marker, IL-10, and the endogenous antioxidants, heme oxygenase (HO)-1, glutamate-cysteine ligase catalytic subunit (GCLC), glutamate-cysteine ligase modifier subunit (GCLM), and NADPH quinone oxidoreductase-1 (NQO1) [108]. The enhancement of the M2 markers and endogenous antioxidants by quercetin was activated by the AMP-activated protein kinase (AMPK) and Akt signaling pathways. Together, the study reported that the quercetin inhibited the effects of M1 polarization, including neuroinflammatory responses, ROS production, and phagocytosis. Moreover, the quercetin enhanced the M2 macrophage polarization and endogenous antioxidant expression in both macrophages and microglia [104]. Naringenin also has potential effects on macrophages. Studies have shown that naringenin can cross the blood–brain barrier, reduce Aβ deposits and restore memory function in transgenic AD mice [109]. Treatment of primary cultured microglia with Aβ_1-42_ significantly promoted M2 microglia polarization and inhibited Aβ_1-42_-induced M1 microglia activation after naringenin administration. Microglia play a key role in brain Aβ clearance through Aβ-degrading enzymes after phagocytosis. After naringenin treatment, these Aβ-degrading enzymes were down-regulated in M1 microglia and up-regulated in M2 microglia. Thus, naringenin increased Aβ-degrading enzymes in M2 microglia, possibly leading to A decrease in Aβ plaques [110].

Toll-like receptors (TLRs) [111] are a class of pattern recognition receptors associated with neuroinflammation, among which TLR4 is a key factor in the regulation of immune response in the process of central nervous system infection and injury. TLR4 is widely distributed in brain microglia and other cells [112]. When TLR4 pathway is specifically recognized by LPS, it will further activate downstream NF-κB, leading to the release of proinflammatory factors, and then induce nerve cell death through apoptosis and other pathways [113]. Among them, Scutellaria baicalensis is often used in clinical treatment of a variety of acute infectious diseases, such as anti-inflammatory, antipyretic, anti-endotoxin, etc. Its baicalein can down-regulate TLR4 protein in microglial BV2 cell inflammatory model, and has anti-neuroinflammation effect [78]. In the LPS-induced inflammatory model of BV2 cells, the cells were significantly activated, and the secretion of proinflammatory factors IL-6, IL-1β and TNF-α increased [114]. Baicalein has obvious inhibitory effect on the inflammatory reaction. When BV2 cells in the resting state are stimulated by various factors, the NF-κB [115] heterodimer P65-P50 in the cytoplasm will be released into the nucleus and bind to the downstream target genes, thus initiating the transcriptional expression of target genes. The results of nuclear translocation of NF-κB P65 by immunofluorescence detection showed that, After LPS stimulation, the expression of NF-κB p65 protein in BV2 cytoplasm was significantly reduced, and most of the NF-κB p65 protein migrated to the nucleus. At the same time, the protein expressions of i-NOS and COX-2 were significantly down-regulated after administration of baicalein, indicating that baicalein inhibited LPS-induced inflammation in BV-2 cells. Administration of baicalein reversed MPTP-induced motor dysfunction, loss of dopaminergic neurons, and pro-inflammatory cytokine elevation. Baicalein also inhibited NLRP3 and caspase-1 activation and suppressed gasdermin D (GSDMD)-dependent pyroptosis. Additionally, baicalein inhibited the activation and proliferation of disease-associated proinflammatory microglia [116].

#### Potential mechanisms of terpenoid compounds in the prevention and treatment of AD

In recent years, natural drugs with few side effects and high safety have been widely used to treat a variety of diseases, including AD. Ginseng has a long history of medicinal use as it improves health and slows the aging process. Ginsenoside Rg1 in the treatment of AD include improvement in Aβ and Tau pathologies [117], regulation of synaptic function and intestinal microflora, and reduction of inflammation, oxidative stress, and apoptosis. The underlying mechanisms mainly involve the regulation of PKC, MAPK, PI3K/Akt, CDK5, GSK-3β, BDNF/TrkB, PKA/CREB, FGF2/Akt, p21WAF1/CIP1, NF-κB, NLRP1, TLR3, and TLR4 signaling pathways [117]. NADPH oxidase (NOXs) is the main enzyme responsible for excessive ROS production in many tissues. Studies have shown that NOX2 promotes aging-related neuronal oxidative stress damage and brain function loss, and the expression of NOX2 is significantly increased in long-term cultured hippocampal neurons. Ginsenoside Rg1, the main active component of ginseng, attenuates H_2_O_2_ -induced neuronal injury by inhibiting NADPH oxidase 2 (NOX2) and nucleotide binding oligomeric enzyme (NOD) -like receptor protein 1(NLRP1) inflammasome activation in hippocampal neurons in vitro [118]. Ginsenoside Rg1 can inhibit NoX2-mediated neuronal oxidative stress and neuroinflammation in APP/PS1 AD mice [119]. The NOD-like receptor family with three pyridine domains (NLRP3) is the most studied inflammasome sensor receptor protein. NLRP3, together with apoptosis-associated spot-like protein (ASC) and caspase-1 precursor protease, is composed of NLRP3 inflammasome [120]. In studies of AD patients and AD transgenic mice, it has also been found that inflammasome-derived ASC can bind to Aβ in the extracellular space of cells and promote Aβ aggregation, leading to the production of downstream inflammatory factors and promoting inflammatory responses. Some studies have shown that ginsenoside Rg1 has anti-inflammatory effects [121, 122]. Ginsenoside Rg1 can inhibit the production of downstream inflammatory factors and enhance the phagocytosis of Aβ in the neuroinflammatory response of AD.

Oleuropein (OLE), a non-toxic penoid glycoside compound, is the main component in olive leaves. Scholars administered olive leaf extract continuously for 3 months through 5xFAD mouse model. Oleoin was found to reduce neuroinflammation mainly by inhibiting NF-κB pathway and inhibiting the activation of NLRP3 inflammasome and RAGE/HMGB1 pathway [123]. In addition, oleuropein pretreatment of SH-SY5Y cells for 24 h could alleviate the cell death induced by Aβ42 and copper-Aβ42. In the transgenic mouse (APPswe/PS1dE9) model, treated mice (OLE) showed significantly reduced amyloid plaque deposition in the cortex and hippocampus compared with control mice [124].

**Fig. 2:**
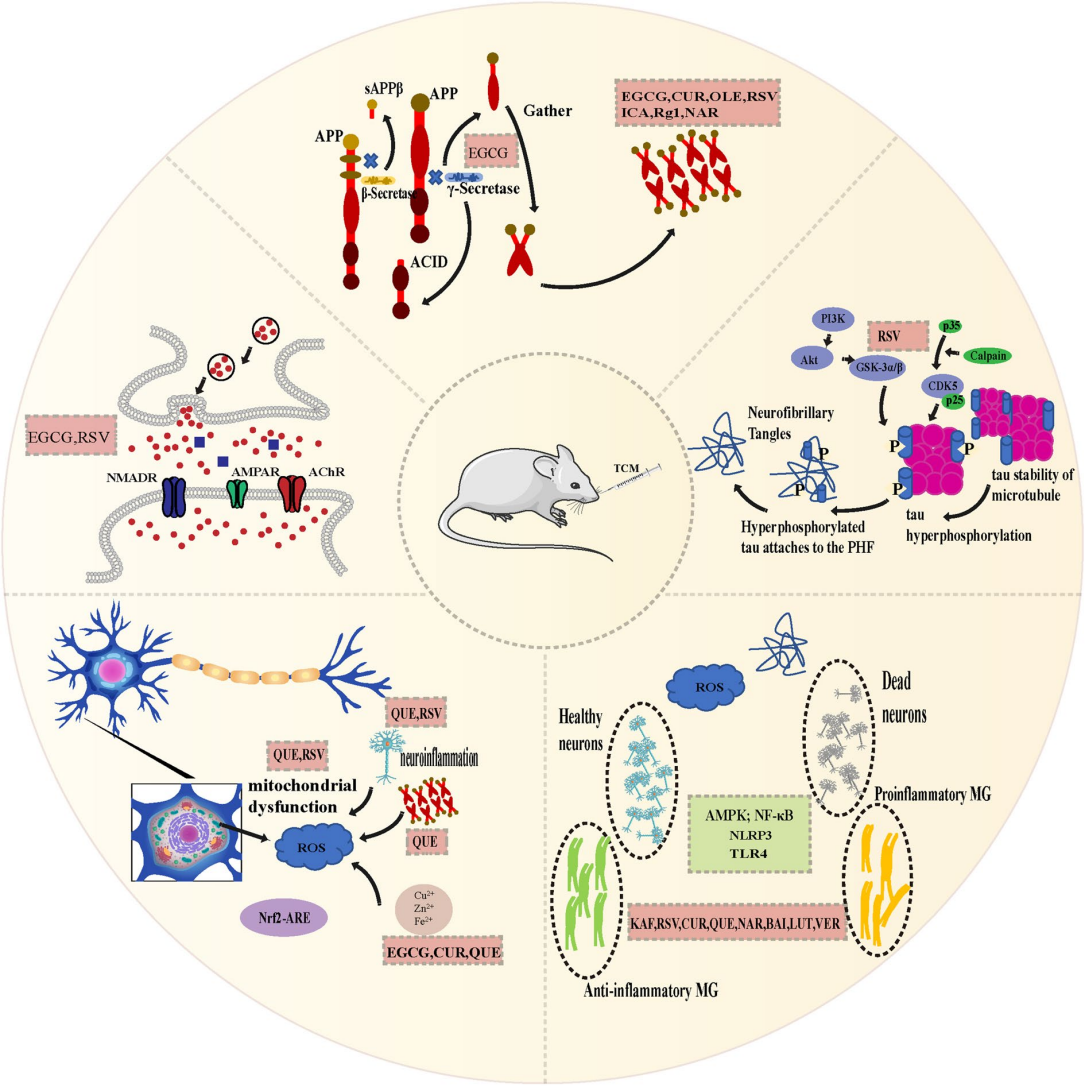
Potential mechanism of traditional Chinese medicine natural products in the treatment of AD. Different types of traditional Chinese medicine monomer compounds play different roles in the process of AD. Epigallocatechin gallate (EGCG), Curcumin (CUR), Oleuropein (OLE), Resveratrol (RSV), Icariin (ICA), Ginsenoside Rg1(Rg1) and Naringenin(NAR) could inhibit the aggregation of Aβ, and resveratrol could prevent the phosphorylation of tau and reduce the level of p-tau. EGCG and RSV inhibited cholinesterase activity. kaempferol(KAF), RSV,CUR,QUE, NAR, Baicalein(BAL), Luteolin(LUT), Verbascoside(VER) inhibited neuroinflammation, Quercetin(QUE),RSV, EGCG reduce oxidative damage and other different pathways play a multi-level, multi-pathway, multi-target drug effect to prevent and control AD

The pathogenesis of AD is complex, including Aβ hypothesis, tau hypothesis, cholinergic hypothesis, inflammation hypothesis, OS hypothesis and cholinergic hypothesis. Current drug therapy for AD can only relieve the symptoms and has a single target, but cannot reverse the course of AD. Chinese herbal compounds, as widely available natural compounds, which have been shown to play a protective role against AD through multiple targets, such as inhibiting Aβ production and aggregation, reducing tau protein hyperphosphorylation and aggregation, regulating cholinergic system, inhibiting neuroinflammation, reducing OS, inducing autophagy and antagonizing NMDAR (Fig. 2). Although more and more studies have been conducted to reveal the mechanism of TCM monomer compounds in the prevention and treatment of AD, the application of the results of in vitro and animal studies to the clinic remains to be further studied.

### Potential mechanism of acupuncture modulating neuroinflammation in the treatment of AD

**Fig. 3:**
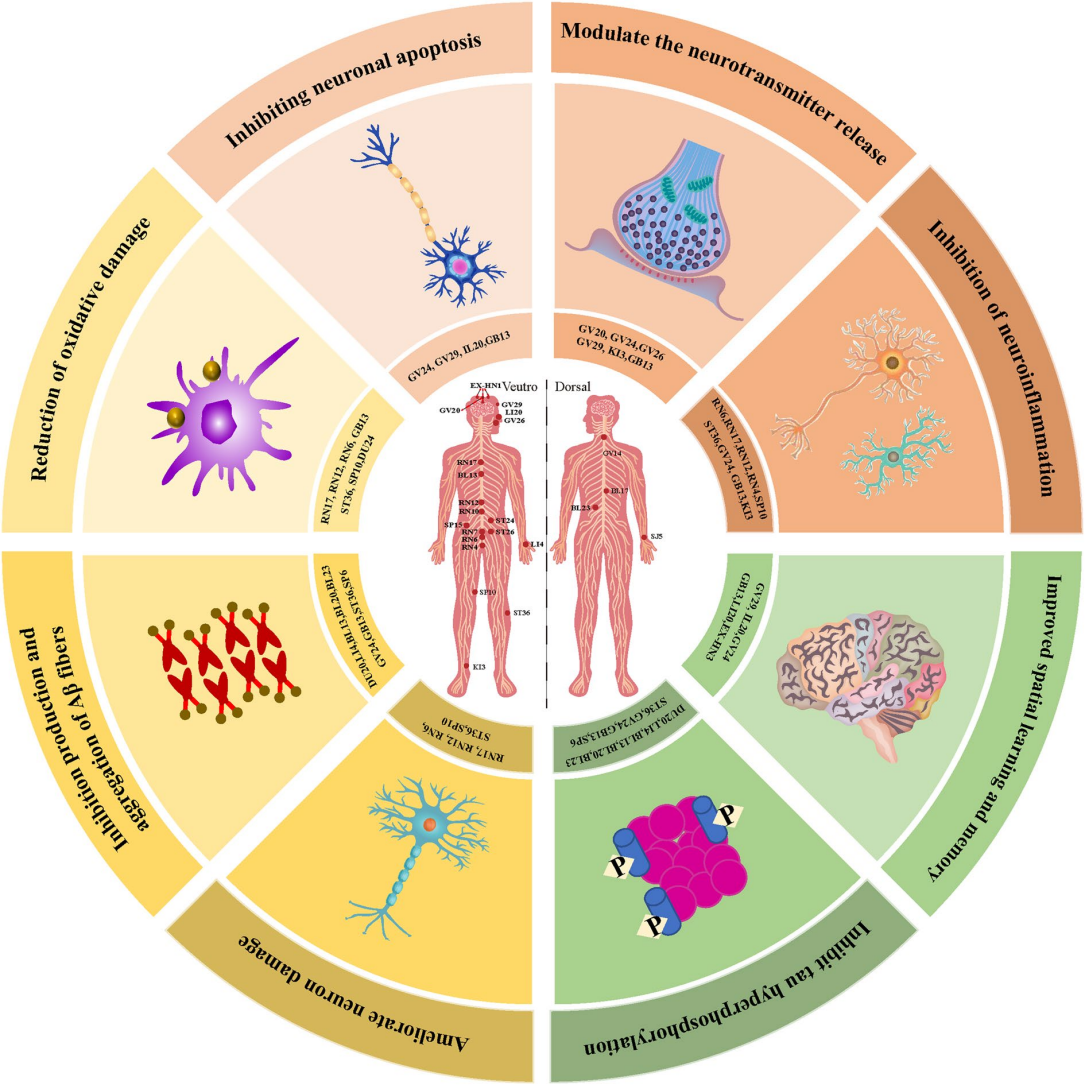
Potential mechanism of acupuncture in the treatment of AD. Acupuncture stimulation of different acupoints can play a role by inhibition production and aggregation of Aβ fibers, inhibit tau hyperphosphorylation, modulate the neurotransmitter release, inhibiting neuroinflammation, reducing oxidative damage and so on

The meridians network the whole body, and the meridians line in the meridians. Acupuncture adjusts the meridians and collaterals, and then adjusts the whole body's meridians [125]. The regulating effect of acupuncture and its meridian and viscera function is more obvious than other meridian. The biological basis of the correlation between meridians acupoints and viscera is mainly segmented interaction of nervous system [126]. Acupuncture plays an important role in the treatment of AD [127], which may improve the daily living ability of AD patients more safely and effectively than drugs, and can enhance the effect of drugs on improving cognitive function. Animal experiments have compared the efficacy of acupuncture at different points in treating animal models of AD, and evaluated the specific effects and neuropathologic mechanisms of acupoint therapy (Table 3, Fig. 3).

**Table 3:** Potential mechanisms of acupuncture ingredients for AD

No.	Intervention	acupoint	Parameter of acupuncture	Model	Signaling pathway	Potential mechanism	**Ref**
1	MA	GV20, GV26, DU29	Two rotations per second for 30 s at each point	senescence-accelerated mouse prone 8 (SAMP8)	-	Iimproved spatial learning and memory ability, the level of glucose metabolism in the brain, and the content of Aβ amyloid in the cortex	[231]
2	EA	GV29, IL20	1.5 mA, 15 Hz	Aβ1-42-induced AD rat model	PI3K/AKT/GSK-3*β*	Rescue the cognitive deficits of A*β*1-42-induced AD rats by improving synaptic plasticity, neuro-apoptosis and neuro-inflammation through enhancing PI3K/AKT/GSK-3*β* signaling pathway	[189]
3	MA	CV17, CV12, CV6, ST36, SP10	Moderate reinforcing-reducing manipulation for 210 s	SAMP8	bFGF, EGF, BDNF	Regulate the cytokine levels associated with survival, pro liferation, and differentiation of NSCs in hippocampal mi croenvironment, to promote the repair of damaged cells, resulting in improved cognitive performance in mice	[232]
4	MA	GV24, GB13	The twisting was performed at a range of 90–180° and a rate of 100–150 times/min	Aβ1-40-induced AD rat model	-	Altering cerebral glucose metabolism (CGM), regulating the distribution of neurotransmitters and enhancing synaptic plasticity	[167]
5	MA	HT7	Twisted/rotated at a frequency of 120–150 times per min for 3 min	IBO-induced AD rat model	-	Improve CGM	[233]
6	MA	CV17, CV12, CV6, ST36, SP10	The needles inserted at each location were stimulated for 30 s and the total treatment time was 210 s	SAMP8	SOD, GSH-Px; superoxide anion, protein carbonyl; Hsp84 and Hsp86 mRNA and protein	Delay brain ageing in SAMP8 mice by reducing oxidative protein damage and promoting Hsp84 and Hsp86 expression	[180]
7	MA	DU20, LI4, BL13, BL20, BL23, ST36, SP6	Acupuncture treatment for 20 min per day	APP/PS1 double-transgenic mice	IL-10, LPS	Regulated the Aβ and tau protein concentration as well as the levels of IL-10 and LPS	[234]
8	EA	LI20,EX-HN3	Current intensity: 1–3 mA, voltage: 1–3 V,80–100 Hz	AD rat model	SOD, GSH-Px	Increase learning-memory ability, decrease MDA content, and increase SOD and GSH-Px activities in the hippocampus	[68]
9	MA	RN17,RN12,RN4,SP10,ST36	Slowly rotated for 30 s every 5 min and lasted for 15 min	SAMP8	PI3K/PDK1/nPKC/Rac 1	Downregulating PI3K/PDK1/nPKC/Rac 1 signaling pathway	[160]
10	MA	CV17, CV12, CV6, ST36, SP10	Each acupuncture point was needled for 30 s, and each non-acupuncture point was needled for 105 s, in order that the total duration of stimulation was 210 s in each group	SAMP8	-	Upregulation of G protein activity and stabilisation of the cellular signal	[235]
11	EA	GV24, GB13	0.3 mA, 2 Hz, 15 min	Aβ-induced AD rat model (5xFAD)	AKT-MAPK1-MTORC1	Inhibited glial cell activation in the prefrontal cortex and hippocampus of 5xFAD, activated TFEB via inhibiting the AKT-MAPK1-MTORC1 pathway, thus promoting ALP in the brains	[143]
12	MA	DU24, GB13	Twisting frequency of 80 ± 5/min, a twisting amplitude of 180° ± 5°, and a sustainable stimulation for 2 min, with 1 min rest; this was repeated for 15 min in total	Aβ1-40-induced AD rat model	neurotransmitter acetylcholine	Related to the role of Zhisanzhen in increasing chAT and Ache activity, decreasing oxidative stress and inhibiting neuronal apoptosis	[236]
13	EA	GV24, GB13	30 Hz, 1.0 mA, 30 min	Aβ1-40-induced AD rat model	-	Decreasing the levels of Aβ, p-tau (s396) and p-tau (s404) in the brain	[165]
14	EA	KI3	15 min, 1 mA, 2 Hz	5xFAD mice model	-	ameliorates cognitive impairment via inhibition of synaptic degeneration and neuroinflammation	[130]
15	MA	ST36	Twirling reinforcing manipulation	CMi rats	NF-κB-p53	Inhibited activation of NF-κB and its downstream target gene p53	[237]
16	MA	CV17, CV12, CV6, ST36, SP10	Rate of twice a second for 30 s at each point	SAMP8	-	Reduced neuron loss in hippocampal regions CA3 and DG	[238]
17	MA	CV17, CV12, CV6, ST36, SP10	Twirling reinforcing manipulation	SAMP8	-	Improve the cognitive impairment by increasing TPI activity, thus correcting the abnormal glycolysis metabolism and maintaining the brain homeostasis and internal environment	[239]
18	EA	GV20, BL23	20 min, 2 Hz,2 mA	Aβ1-40-induced AD rat model	PPAR-γ; p-p38MAPK	activation of PPAR-γ and inhibition of p-p38MAPK expression	[240]
19	MA	ST36	The twisting was performed within the range of 90–180° at a rate of 60–90 times/min	AD rat model	-	Increase blood perfusion and glycol metabolism in certain brain areas	[241]
20	EA	GV20,GV26,GV29	20 min, 2 Hz,1 mA	APP/PS1 mice	-	Enhancing glucose metabolism and inhibiting inflammation-mediated Aβ deposition	[242]
21	EA	GV20, KI1	30 min, 2/15 Hz,1 mA	Aβ1-42-induced AD rat model	NOX2	Alleviation of neuronal injury and inhibition of NOX2-related oxidative stress	[181]
22	EA	GV20,GV26,GV29	2 Hz,1 mA	SAMP8	AQP4	Reduce Aβ accumulation	[169]
23	EA	GV20,GV29	2 Hz,0.1 mA	SAMP8	TREM2	Upregulate TREM2 expression in the hippocampus	[243]
24	EA	GV20, BL23	20 Hz,2 mA	Aβ1-40-induced AD rat model	LC3II/LC3I ratios, Beclin-1	Reduces neuronal apoptosis, enhances degradation of Aβ, and improves learning/memory	[244]
25	EA	DU24, DU20	30 min, 1/20 Hz,1 mA	APP/PS1 mice		Regulate microglial polarization and decrease Aβ plaque	[245]
26	EA	DU24, DU20	30 min, 2/20 Hz,1 mA	5xFAD mice	MS/VDB-DG cholinergic neural circuit	Improve the early pattern separation impairment by activating the MS/VDB-DG cholinergic neural circuit	[246]
27	EA	DU20, BL23	15 min, 2 Hz,2 mA	APP/PS1 mice	-	Improve the ability of learning, memory and spatial exploration, and reduce the deposition of SPs in brain of AD model mice, and reduce the expressions of APP and BACE1, increase the expression of IDE protein	[247]
28	EA	GV20, GV29	2 Hz,0.1 mA	SAMP8	IL-1β, IL-6, TNF-α	inhibit the peripheral and central nerve system inflammatory response by balancing the gut microbiota	[188]
29	MA	GV20, GV29	Each needle was rotated bidirectionally within 90° at a speed of 180°/s	SAMP8	-	Increase in CBF in the prefrontal lobe and hippocampu	[248]
30	EA	GV20, BL23	20 min, 2/3/50 Hz,1 mA	Aβ1-42-induced AD rat model	GSK-3β	Inhibition of GSK-3β activity	[176]
31	EA	GV29, LI20	10 min, 15 Hz,1.5 mA	SAMP8	p38MAPK	Inhibited the phosphorylation of p38MAPK and the excessive activation of microglia (MG) in the hippocampus	[67]

#### Acupuncture reduces neuroinflammation by inhibiting the activation of MG and astrocytes

Neuroinflammation is an immune response activated by glial cells in the CNS. It mainly occurs in response to stimuli, such as nerve injury, infection, and toxins, or in response to autoimmunity. Neuroinflammation is closely associated with the progression of neurodegenerative diseases, including AD, PD, ALS, and multiple sclerosis [37]. Recent studies have indicated that neuroinflammation has emerged as the third prominent pathological feature, following Aβ deposition and NFTs in AD. Inflammatory responses assume a pivotal role in both the onset and progression of AD [128, 129].

Studies have shown that Aβ can bind to receptors on the surface of microglia and astrocytes, trigger the release of inflammatory cytokines and chemokines, lead to the occurrence of chronic inflammation, mediate neuroinflammation and neurotoxicity, and impair cognitive function. Numerous studies have demonstrated that EA treatment can inhibit the activation of microglia and astrocytes in the hippocampus of AD patients, and reduce the deposition of Aβ [130]. The anti-inflammatory effect is related to the increase of the levels of anti-inflammatory cytokines (e.g., interleukin-2 (IL-2), IL-4, IL-10, IL-13, etc.) [23, 131] and the decrease of the levels of pro-inflammatory cytokines (e.g., tumor necrosis factor-α (TNF-α), IL-1β, and IL-6) [132, 133]. Acupuncture exhibited to significantly improve the working memory and synaptic plasticity of AD mice by inhibiting the phosphorylation of p38MAPK and the over-activation of MG cells in the hippocampus [67], as well as reduction of synaptic ultrastructural degradation in AD mice [134].

In the CNS, triggering receptor expressed on myeloid cells 2 (TREM2) is mainly expressed on the surface of microglia cells, mediating the proliferation, differentiation, survival, autophagy, and the expression levels of inflammatory factors in microglia cells, highlighting its crucial role in AD pathogenesis. Besides, Aβ was recently characterized as a ligand of TREM2, as it could directly bind TREM2 and activate TREM2 signaling pathway [135]. In vivo*,* TREM2 expression level is mainly upregulated under pathological conditions. For instance, increased expression level of TREM2 have been identified in AD patients [136] and in Aβ and tau pathological mouse models [137]. Overexpression of TREM2 is thought to be related to the recruitment of microglia to Aβ plaques [138]. As senescence accelerated mouse prone 8 (SAMP8) mice age, they exhibited accelerated cognitive decline, accompanied by pathological alterations in the CNS, including the cortex and hippocampus. These age-related changes align with the parameters monitored in the experiment. SAMP8 mice serve as valuable models for investigating the mechanisms underlying aging, cognitive function, and dysfunction, as well as for assessing potential anti-aging and cognitive enhancement interventions. Jiang et al.’s research revealed that EA could enhance TREM2 protein level in SAMP8 mice, thereby improving spatial learning and memory abilities, enhancing hippocampal neuronal morphology, and reducing the expression level of Aβ1-42 protein [139]. Thus, acupuncture may affect the pathological process of AD by regulating neuroinflammation in the brain through MG, AS, and TREM2.

Many studies have found that there is an interaction between neuroinflammation and autophagy in the pathogenesis of neurodegenerative diseases. LPS-induced neuroinflammation can cause autophagic damage through ATG gene imbalance. Autophagy, as a conservative catabolic process, can degrade defective proteins or organelles in lysosomes and recycle important components in eukaryotic cells. Under normal circumstances, it can have a protective effect, but abnormal autophagy may lead to cell death. Increasing evidence suggests that dysfunctional autophagy is an important trigger for AD [140]. Research indicates that autophagy is involved in the pathogenesis of AD, including Aβ metabolism, tau pathology, synaptic function, and mitochondrial dysfunction. Therefore, autophagy and neuroinflammation are expected to be used in the treatment of AD [141]. Chenglong Xie [142] used computer-aided drug screening technology to identify 18 small molecules that can act as mitochondrial autophagy inducers, increasing the survival and function of glutamate and acetylcholine neurons, eliminating amyloid-β and tau pathology, and improving the pathological symptoms of AD. Zheng Xiaoyan's [143] research has demonstrated that electroacupuncture can significantly improve cognitive dysfunction in 5xFAD transgenic mice expressing Aβ. Electroacupuncture achieves this by inhibiting the AKT-MAPK1-MTORC1 pathway in the prefrontal cortex and hippocampus, activating TFEB, and promoting the autophagic degradation of APP/Aβ. Lin Wenjia's [144] research found that the acupuncture points GV24 and bilateral GB13 can alleviate memory impairment related to Alzheimer's disease by promoting the autophagic clearance of Aβ and NLRP3 inflammasomes mediated by TFEB/TFE3.Although the relationship between mitochondrial autophagy and the pathogenesis of AD is still under investigation, acupuncture's regulation of mitochondrial autophagy-mediated clearance of dysfunctional mitochondria has shown potential for intervening in the treatment of AD.

#### Acupuncture reduces neuroinflammation and Aβ protein production by inhibiting inflammatory response

##### TLR4/NF-κB/NLRP3 signaling pathway

Inflammasome plays a key role in neuroinflammatory pathways, and it may be a target for AD therapy. Inflammasomes are an important component of innate immunity. They are multiprotein complexes, consisting of caspase, apoptosis-related spot-like proteins (ASCs), and intracytoplasmic pattern recognition receptors (PRRs) that recognize pathogen-associated molecular patterns (PAMPs) or damage-associated molecular patterns (DAMPs), recruit and activate pro-inflammatory protease Caspase-1, and activated Caspase-1 cleaves IL-13 and IL-18 precursors to produce corresponding mature cytokines [145]. Five major inflammasome types have been found, including NLRP1, NLRP3, NLRC4, IPAF, and AIM2 inflammasomes. Jing Jiang et al. [146] studied the relationship between EA and the expression level of NLRP-3 inflammasome in the hippocampus of AD animal models, and they found that EA could inhibit the inflammatory response in the hippocampus of SAMP8 mice. In addition, the possible mechanism of EA is to reduce the expression levels of IL-1β and NLRP3 inflammasomes-related proteins. Other studies have also demonstrated that acupuncture can reregulate the expression levels and activities of inflammasomes, reduce inflammatory response in brain, and improve memory deficit and synaptic plasticity. Chuan He et al. [147] found that acupuncture pretreatment could inhibit the protein expression levels of NLRP3, Caspase-1, and IL-1β, and reduce the amount of activated MG in the hippocampus of rats. Ning Ding et al. [148] showed that MA could negatively regulate the NLRP3/Caspase-1 pathway in the hippocampus of AD mice. Kun Li et al. [149] reported that EA intervention in Baihui (GV 20) and Shenting (GV 24) inhibited the elevation of the expression levels of NLRP3, Caspase-1, IL-1β, and IL-18, improved memory deficits, and impaired synaptic plasticity. APP/PS1 double transgenic mice serve as valuable models for AD research, expressing mutant human presenilin DeltaE9 and a fusion gene of human mouse preamyloid protein APPswe under the regulation of mouse prion promoters. The DeltaE9 mutation, arising from the ninth exon’s deletion in the human presenilin gene is associated with early-onset AD. Dong-Mei Liao's investigation [150] has revealed that EA could effectively mitigate the overactivation of the TLR4/NF-κB/NLRP3 signaling pathway, thus ameliorating CNS inflammation and enhancing cognitive function in APP/PS1 mice. These findings suggest that acupuncture may exert its effects through modulation of the TLR4/NF-κB signaling pathway to suppress inflammasome activation. Furthermore, acupuncture has shown to reduce Aβ protein accumulation in the hippocampus by inhibiting inflammasome activation. Ting Zhang’s research [151] demonstrated that EA could reduce Aβ production in the hippocampal tissue of SAMP8 mice, mitigate neuronal apoptosis and damage, and inhibit NLRP1 inflammasome activation in these mice.

##### PI3K/Akt signaling pathway

Yuan Wang et al.’ research [152] revealed that Olfactory Three-Needle acupuncture significantly enhanced the PI3K/AKT signaling pathway, leading to the phosphorylation inactivation of GSK-3β in AD. This effect could improve synaptic plasticity and neuronal survival. In Xinyu Huang et al.’s study [153], EA intervention led to significant reductions in Aβ and total tau protein levels in the hippocampus, as well as body weight. These changes were associated with modulation of the PI3K/Akt signaling pathway. Yinshan Tang et al.’s findings [154] indicated that EA could reverse cognitive deficits in AD model mice (APP/PS1) by inhibiting the JNK signaling pathway and modulating apoptotic signals. Zhitao Hou et al. [155] demonstrated that 10 HZ EA effectively alleviated learning and memory impairment in 7-month-old SAMP8 mice. This intervention reduced pathological hippocampal damage, enhanced synaptic structure, improved synaptic transmission, and regulated the expression levels of proteins related to the cAMP/PKA/CREB signaling pathway. Yan-Jun Wu et al.’s results [156] showed that EA effectively regulated the expression levels of proteins related to the PI3K/GSK3α signaling pathway in the hippocampal tissue of AD mice, reducing the formation and deposition of senile plaques (SPs). Dong Weiguo et al.’s research [157, 158] indicated that EA could mitigate synaptic loss, increase the expression levels of SYN and PSD95, and inhibit AMPK activation and eEF2K activity. These effects could be associated with the inhibition of the AMPK/eEF2K/eEF2 signaling pathway. Jian-Qiao Fang et al. [159] reported the decreased expression levels of p-p38 MAPK protein and IL-1β mRNA in the frontal lobe and hippocampus of AD rats following EA intervention. Li et al. found that acupuncture at RN17, RN12, RN4, SP10, and ST36 points significantly improved the cognitive function of SAMP8 mice, reduced inflammation, and attenuated nuclear damage by downregulation of the PI3K/PDK1/nPKC/Rac 1 signaling pathway [160]. Studies have demonstrated that the low activity of glucose transporters and insufficient glucose intake in the brain tissues of AD patients and animals could lead to glucose metabolism disorders in the brain, and the binding of advanced glycosylation terminal products with receptors in neuroinflammatory plaques and NFTs could further induce OS and promote the pathological process of AD.

##### GSK-3β signaling pathway

Acupuncture reduces neuroinflammation by inhibiting the production of Aβ protein and phosphorylation of Tan protein. NFT is the main pathological feature of AD. The hyperphosphorylated tau protein is an important component of NFT and one of the main influential factors of AD. Inhibiting tau protein phosphorylation is a crucial method to prevent and treat AD. GSK-3β is a serine/threonine protein kinase, which is involved in the regulation of various intracellular signal transduction pathways, and it plays a major role in gene expression, apoptosis, and neuronal plasticity [161] [58]. GSK-3β has two isoforms, α and β, which share 98% identity in the catalytic region, while differ slightly in the N-terminus and C-terminus. The activity of GSK-3β is regulated by phosphorylation level, and the activation of GSK-3β can inhibit some transcription factors that promote neuronal survival, while its inactivation can promote neuronal survival and improve the stability of cell structure [58]. Activation of Akt promotes phosphorylation of GSK-3β in the CNS. At resting state, GSK-3β can downregulate β-catenin level and inhibit neuronal proliferation, differentiation, and migration. Moreover,, it is important to consider the expression levels of Bcl-2 and Bax [162], downstream proteins of Akt, as they can influence the expression level of Caspase-3. In AD pathophysiology, GSK-3β plays a pivotal role, impacting various disease aspects, including tau phosphorylation, Aβ production, memory, neurogenesis, synaptic function, and serving as a potential therapeutic target for AD. Chao Yu et al.’s research demonstrated that prophylactic EA at GV20 and BL23 acupoints improved synaptic and neuronal microtubule damage in D-Galactose-induced AD rats. The underlying mechanism was associated with the inhibition of GSK3β/mTOR pathway activity, resulting in the reduced tau phosphorylation and enhanced autophagy activity [163]. Anping Xu et al. [164] demonstrated that EA treatment significantly enhanced cognitive ability and hippocampal glucose uptake in APP/PS1 mice. The phosphorylation of tau protein is inhibited by inducing AKT and GSK3β phosphorylation. Therefore, the AKT/GSK3β signaling pathway may play an irreplaceable role in the regulation process. Yang et al. [165] studied the effects of acupuncture on cognitive function and the mechanism of treatment, and they found that EA at GV24 and bilateral GB13 points could reduce the levels of Aβ, p-tau (s396), and p-tau (s404) in the brain. Wang et al. [166] found that EA could reduce the overexpression of phosphorylated tau protein (Ser199, Ser202) in hippocampus of rats to improve cognitive function in AD rats.

#### Acupuncture reduces neuroinflammation by improving synaptic plasticity

Aβ deposition can lead to the onset of AD, and reducing the accumulation of Aβ in the brain can delay or alleviate AD symptoms. A large number of studies have shown that Aβ is a common pathway induced by various causes of AD, and it plays a noticeable role in the formation and development of AD. In an Aβ 1–40-induced AD rat model, acupuncture administered at GV24 and bilateral GB13 revealed notable changes in cerebral glucose metabolism (CGM) within the hypothalamus, thalamus, and brain stem, as indicated by positron emission tomography (PET). These changes suggest that acupuncture may enhance the learning and memory capabilities of AD rats [167]. Additionally, EA treatment led to a reduction in Aβ deposition mediated by MG, aligning with decreased amyloid precursor protein level [130]. Lin-Mei Wang et al. [168] showed that EA treatment at GV20 and BL23 effectively reduced inflammatory response and Aβ level in APP/PS1 mice. EA also enhanced the autophagic state, improved lymphatic system clearance ability [169], and reduce intracellular Aβ [170] to improve the learning and memory abilities of mice. Studies [171–173] have shown that EA could reduce the accumulation of Aβ in the hippocampus of APP/PS1 mice and promote neurogenesis, and the mechanism may be related to energy metabolism [174] and synaptic regulation [175]. The 5xFAD mouse model, generated by combining three human APP mutants with two PS1 mutations, exhibited early-onset amyloidosis, cognitive impairment, and neuronal loss. EA at GV20 and BL23 [176] enhanced hippocampal synaptic transmission, aiding in synaptic injury recovery. Furthermore, EA at GV20, Dazhui (DU 14), and BL23 improved synaptic ultrastructure, while acupuncture at RN17, RN12, RN6, ST36, and SP10 enhanced dendritic structure [177]. High-frequency EA (50 Hz) proved more effective than low-frequency (2 Hz) or medium-frequency (30 Hz) EA [176]. Mudan Cai [130] was used in 5xFAD mice, bilateral EA treatment at KI3 points significantly enhanced working memory and synaptic plasticity, reduced neuroinflammation, and mitigated synaptic ultrastructure degradation by bolstering synaptic functions. Furthermore, Xiaoyan Zheng's [143] research also shows that three-needle EA intervening at the GV24 and bilateral GB13 acupoints with EA, spatial learning and fear memory in 5xFAD mice were significantly improved. This treatment approach also resulted in the decreased levels of APP, its C-terminal fragment (CTFs), and Aβ deposits, and inhibited glial cell activation in the prefrontal cortex and hippocampus of 5xFAD. Yu et al. [176] and Yimin Jiang et al. [178] assessed the improvement effect of acupuncture at different frequencies on AD mice, and they found that high-frequency EA exhibited promising results, effectively inhibiting the activity of GSK-3β in the presence of Aβ1-42. This inhibition led to a reduction in learning and memory impairments induced by Aβ1-42 and provided protection against damage to synaptic ultrastructure.

Ran Ma et al. [179] investigated the effects of EA and MA on the learning and memory abilities, the ultrastructural changes of neurons, and the downregulation of CDK5 and tau proteins in the hippocampus of SAMP8 mice. Yang et al. [179] demonstrated that the expression levels of phosphorylated tau protein and tau mRNA in the hippocampus of the 3-month-old EA group decreased compared with that of the 9-month-old EA group, indicating that early EA intervention could more effectively improve the learning and memory abilities of SAMP8 mice and inhibit the phosphorylation of tau protein in the hippocampus.

#### Acupuncture reduces neuroinflammation by alleviating OS

The production of free radicals is an important factor causing OS, and OS plays a noticeable role in the early onset of AD. Excessive OS can not only cause neural cell death, but also lead to brain tissue damage. Therefore, inhibiting OS may be an important measure to prevent AD. Chang et al. [180] reported that after undergoing acupuncture treatment, the cognitive function of mice was improved, the number of neurons increased, the levels of superoxide dismutase (SOD) and glutathione peroxidase (GSH-Px) were elevated, and the levels of superoxide anion and protein carbonyl were reduced. Therefore, acupuncture may delay the brain aging of SAMP8 mice by alleviating OS. Wu et al. [181] found that EA treatment significantly restored total antioxidant capacity (T-AOC) and attenuated the abnormal increase in levels of ROS, MDA and 8-hydroxy-2-deoxyguanosine (8-OH-dG). It can effectively improve the hippocampal neuron damage and counteract the abnormal increase of NOX2 level in AD rats. Through precise needling of DU24 and GB13 acupoints, Cai et al. [130] demonstrated significant enhancements in behavior, a reduction in OS, the elevated level of the neurotransmitter acetylcholine, diminished apoptosis of hippocampal neurons, and a decrease in the expression levels of genes and proteins associated with apoptosis.

#### Acupuncture can increase the abundance of intestinal flora and inhibit neuroinflammatory response

Inflammatory response is closely associated with the imbalance of intestinal microbial community [182]. Traditionally, AD research primarily centered on the brain. However, in recent years, there was a shift towards investigating the role of GM in this context. Extensive studies on GM’s influence on the nervous system have revealed substantial disparities in GM composition and metabolite content between AD patients and the general population [183]. Moreover, interventions involving the reconstruction of intestinal flora have demonstrated the capability to reduce neuroinflammation in the brain and significantly enhance cognitive function in patients with AD [184–186]. These findings suggest that the microbial-gut-brain axis is an important pathologic pathway, influencing the occurrence and development of AD [187]. Yue Zhang et al. [12] evaluated the effects of MA on BBB dysfunction in APP/PS1 mice, and they demonstrated that MA could positively regulate intestinal flora and BBB dysfunction, and reduce the levels of TNF-α and IL-1β. Jing Jiang et al. [188] reported that EA could balance the number and composition of intestinal microbiota in SAMP8 mice and significantly reduce the expression levels of IL-1β, IL-6, and TNF-α in serum and hippocampal tissue. Therefore, EA can improve the abundance of intestinal flora and inhibit neuroinflammatory response.

Collectively, the acupuncture can not only inhibit production and aggregation of Aβ protein and phosphorylation of tau protein, but also can inhibit neuroinflammation, promote neurogenesis, regulate synaptic plasticity of the CNS [189], alleviate OS, improve disorders of intestinal flora, modulate brain glucose metabolism, and exert an effect of neuroprotection on the CNS cells.

## Conclusions and perspectives

Neurodegenerative diseases are characterized by a progressive loss of neuronal structure and function and an overactive inflammatory response. An increasing number of studies have shown that the pathological mechanisms of neurodegenerative diseases are complex and poorly understood. The onset of AD is slow or insidious, mainly manifested by cognitive decline, psychiatric symptoms and behavioral disorders, and gradual decline in the ability of daily living. Divided into three stages according to the degree of cognitive and physical deterioration, including mild, moderate and severe dementia, Aβ [190, 191] plaque deposition usually leads to the accumulation of misfolded proteins and the production of reactive oxygen species, causing oxidative stress [192], resulting in membrane damage, mitochondrial dysfunction [193–195], and neurotrophic damage [196–198],At the same time, it can induce neuroinflammatory reaction [199], microglia activation, cytokine release and astrogliosis cascade reaction, thereby producing cytokines and triggering a series of inflammatory reactions, leading to synaptic dysfunction and inducing neurotoxic effects (Fig. 1). Due to the accurate diagnosis is difficult in preclinical AD, treat the patient began to appear the symptom of mild cognitive dysfunction period, disease has entered the early and mid-mostly, with Aβ again just at this time of treatment is to control the disease, such as so on single targets, such as Aβ, Tau protein failure, new drug research and development of resistance to the AD In addition to Aβ and Tau proteins, the CD33 and TREM2 genes involved in the regulation of glial cell function have been targeted to develop new anti-AD drugs. Although many drugs are in clinical trials, only a small fraction of these drugs have been successfully developed and approved for the treatment of neurodegenerative diseases [200, 201]. Although neuroinflammation may not be the trigger of neurodegenerative diseases, persistent inflammation can create a vicious cycle between neuronal lesions, leading to more neuronal death. Therefore, our treatment needs to focus on inhibiting microglial activation, proinflammatory cytokines and excessive production of oxidative stress.

According to TCM theory, the brain is the "house of the primitive God", which controls the life and spiritual activities of human beings, stores the essence and connects with the pulp, and is the place of life consciousness. Encephalopathy has been a serious threat to human health since ancient times. The multi-target strategy of TCM in the treatment of encephalopathy with "tonic deficiency", "tonic stasis" and "eliminate phlegm" as the treatment principle can provide new ideas for drug research and development of complex diseases such as AD. Modern pharmacological studies have found that many traditional Chinese medicine ingredients, such as tonifying, activating blood circulation and removing blood stasis, resolving phlegm, and awakening the mind, can inhibit inflammation in vivo and in vitro, and can be used as candidate drugs for the treatment of AD. It plays a role in inhibiting Aβ production and aggregation, reducing p-tau protein level, inhibiting neuroinflammation, alleviating oxidative damage and inhibiting cholinesterase activity. Through cell and animal models, a large number of researchers have found that traditional Chinese medicine compounds, monomer compounds or other traditional Chinese medicine therapies, such as acupuncture and moxibustion, have shown good efficacy, which can slow down the process of AD by regulating microglia function, inhibiting inflammation, improving oxidative stress and nutritional nerve. However, studies have shown that malabsorption, rapid metabolism and systemic elimination, inefficient drug delivery systems, and selective permeability across the BBB [202] are also serious problems, which largely limit the bioavailability and neuroprotective effects of TCM in neurodegenerative diseases. With the development of pharmaceutical technology, nanotechnology [203] has been used to increase the permeability of the BBB and improve the bioavailability of drugs. Compared with resveratrol alone, liposome resveratrol [204, 205] has more obvious antioxidant, free radical scavenging and ROS production reduction effect. Therefore, how to improve the absorption and stability of TCM, structure and formulation improvements, and more technologies and strategies for combination therapy are being developed, which provide more opportunities for TCM treatment of AD.

Acupuncture and moxibustion in the treatment of AD has the advantages of early, safe, effective [206] and benign bidirectional adjustment [207]. Acupuncture stimulation of different acupoints can play a role by inhibiting the production and aggregation of Aβ, preventing excessive phosphorylation and aggregation of tau protein, regulating the cholinergic system, inhibiting neuroinflammation, reducing oxidative damage and so on.In recent years, brain imaging technologies, such as functional magnetic resonance imaging and positron emission tomography, have been used to assess brain responses to acupuncture in a dynamic, visual, and objective way. These techniques are frequently used to explore neurological mechanisms of responses to acupuncture in AD and provide neuroimaging evidence as well as starting points to elucidate the possible mechanisms [208]. To further study the mechanism of acupuncture on AD, scholars combine it with acupuncture and moxibustion theory and put forward different acupuncture and moxibustion rules and acupoint selection prescriptions. MA and EA [62] therapy still occupy an important position, and the curative effect is certain. Body acupuncture acupoints are mainly Du meridian, heart meridian, pericardial meridian and kidney meridian, and the reinforcing and reducing technique is clear. The intensity, amplitude and frequency of electroacupuncture stimulation also have clear parameter range. Some scholars have proved that acupuncture combined with Chinese herbal medicine is more effective and safer in the treatment of diabetic neuropathy [209]. Therefore, the combination of acupuncture and medicine is expected to become a new treatment for AD, and the organic combination of basic and clinical research needs to be further strengthened, improve research level, perfect the unified diagnosis and curative effect evaluation standard, to gradually achieve objective research, standardization, standardization, for a variety of different stitch to strengthen clinical design scientific, rigor, at the same time, It is necessary to continue to strengthen the organic combination of clinical research and experimental research. In experimental research, it is necessary to analyze not only the influence of acupuncture and moxibustion on various experimental indicators, but also further study and analyze the influence of various experimental indicators on the clinical effect of acupuncture and moxibustion, so that experimental research can better serve the clinical practice. Finally, in order to further prove the efficacy and safety of TCM in the treatment of AD, more randomized controlled trials with high accuracy, clinical safety, rigorous design and large sample size should be carried out, and the mechanism of compatibility principle should be further explored.

All in all, the purpose of this review is to scientific and systematic evaluation of TCM in the role of AD, in combination with the existing clinical and preclinical evidence, a comprehensive discussion and shows the TCM treatment for AD curative effect and potential mechanism. In short, the application of modern Chinese medicine theory and modern scientific and technological means to deeply explore the pathogenesis of neurodegenerative diseases, and provide more reliable evidence for the treatment of AD with Chinese medicine.

## Data Availability

No data was used for the research described in the article.

## Abbreviations

AD: Alzheimer’s disease
ADL: Activity of daily living
AS: Astrocytes
ALS: Amyotrophic lateral sclerosis
Aβ: Beta-amyloid
APP: Aβ precursor protein
Ach: Acetylcholine
AChE: Acetylcholinesterase
ADAS-Cog: AD Cognitive Assessment
AlCl: Aluminum chloride
AMPK: AMP-activated protein kinase
ASCs: Apoptosis-related spot-like proteins
BBB: Blood–brain barrier
BSR: Bushen Recipe
CBQ: Caregiver burden questionnaire
CNS: Central nervors system
CGM: Cerebral glucose metabolism
CGRP: Calcitonin gene-related peptide
ChAT: Acetylcholintransferase
CTFs: C-terminal fragment
DAMPs: Damage-associated molecular patterns
DTD: Di-tan decoction
d-gal: D-galactose
ET: Endothelin
EA: Electro-acupuncture
FDA: Food and Drug Administration
GCLC: Glutamate-cysteine ligase catalytic
GCLM: Glutamate-cysteine ligase modifier
GFAP: Glial fibrillary acidic protein
GSH-Px: Glutathione peroxidase
HO: Heme oxygenase
HI: Hippocampus
IL: Interleukin
iNOS: Inducible nitric oxide synthase
LPS: Lipopolysaccharides
MA: Manual acupuncture
MCA: Middle cerebral artery
MCI: Mild cognitive impairment
MDA: Malondialdehyde
MG: Microglia
MoCA: Montreal cognitive assessment
MS: Multiple sclerosis,
MMSE: Mini Mental State Inventory
NMDA: N-methyl-D-aspartate
NFTs: Neurofibrillary tangles
NLRP3: NOD-like receptor family with three pyridine domains
NQO1: NADPH quinone oxidoreductase-1
NO: Nitric oxide
OS: Oxidative Stress
OLE: Oleuropein
PD: Parkinson's disease
PHFs: Paired helical filaments
PRRs: Pattern recognition receptors
PAMPs: Pathogen-associated molecular patterns
PET: Positron emission tomography
PFC: Prefrontal cortex
PRRs: Pattern recognition receptors
PAMPs: Pathogen-associated molecular patterns
PET: Positron emission tomography
ROS: Reactive oxygen species
RCTs: Randomized controlled trials
rs-fMRI: Resting-state fMRI
SCI: Spinal cord injury
SOD: Superoxide dismutase
SHRs: Spontaneously hypertensive rats
SFs: Straight filaments
SPs: Senile plaques
SAMP8: Senescence accelerated mouse prone 8
Sirt1: Silent type information regulation 2 homolog1
TCM: Traditional Chinese medicine
TLRs: Toll-like receptors
TREM2: Triggering receptor expressed on myeloid cells 2
T-AOC: Total antioxidant capacity
TNF-α: Tumor necrosis factor-α
TXR: Tiaoxin Recipe
VD: Vascular dementia
WHO: World Health Organization
8-OH-dG: 8-Hydroxy-2-deoxyguanosine
